# Identification of Diagnostic Biomarkers for Compensatory Liver Cirrhosis Based on Gut Microbiota and Urine Metabolomics Analyses

**DOI:** 10.1007/s12033-023-00922-9

**Published:** 2023-10-24

**Authors:** Yingjun Chen, Shaoxian Chen, Chandi Xu, Li Yu, Shanshan Chu, Jianzhi Bao, Jinwei Wang, Junwei Wang

**Affiliations:** 1 Department of Infectious Diseases, Tiantai People’s Hospital of Zhejiang Province, Taizhou, 317200 People’s Republic of China; 2 Department of General Medicine, Tiantai People’s Hospital of Zhejiang Province, Taizhou, 317200 People’s Republic of China

**Keywords:** Compensatory liver cirrhosis, Gut microbiota, Urine metabolomics, Early diagnosis, Inflammation

## Abstract

Liver cirrhosis is one of the most prevalent chronic liver disorders with high mortality. We aimed to explore changed gut microbiome and urine metabolome in compensatory liver cirrhosis (CLC) patients, thus providing novel diagnostic biomarkers for CLC. Forty fecal samples from healthy volunteers (control: 19) and CLC patients (patient: 21) were undertaken 16S rDNA sequencing. Chromatography-mass spectrometry was performed on 40 urine samples (20 controls and 20 patients). Microbiome and metabolome data were separately analyzed using corresponding bioinformatics approaches. The diagnostic model was constructed using the least absolute shrinkage and selection operator regression. The optimal diagnostic model was determined by five-fold cross-validation. Pearson correlation analysis was applied to clarify the relations among the diagnostic markers. 16S rDNA sequencing analyses showed changed overall alpha diversity and beta diversity in patient samples compared with those of controls. Similarly, we identified 841 changed metabolites. Pathway analysis revealed that the differential metabolites were mainly associated with pathways, such as tryptophan metabolism, purine metabolism, and steroid hormone biosynthesis. A 9-maker diagnostic model for CLC was determined, including 7 microorganisms and 2 metabolites. In this model, there were multiple correlations between microorganisms and metabolites. *Subdoligranulum*, *Agathobacter*, *norank_f_Eubacterium_coprostanoligenes_group*, *Butyricicoccus*, *Lachnospiraceae_UCG_004*, and L-2,3-Dihydrodipicolinate were elevated in CLC patients, whereas *Blautia*, *Monoglobus*, and 5-Acetamidovalerate were reduced. A novel diagnostic model for CLC was constructed and verified to be reliable, which provides new strategies for the diagnosis and treatment of CLC.

**Supplementary Information:**

The online version contains supplementary material available at 10.1007/s12033-023-00922-9.

## Introduction

Liver cirrhosis (LC) is one of the most frequent chronic liver diseases worldwide and a dominant cause of death, accounting for 2% of global mortalities yearly [1, 2]. The main characteristics of LC are altered liver architecture induced by regenerative nodules and diffuse fibrosis, resulting in intrahepatic vascular change, portal hypertension, and secondary liver failure [3]. Furthermore, LC is a risk factor for hepatocellular carcinoma, and the morbidity of hepatocellular carcinoma in LC patients is 2–7% [4]. The progression of LC can be split into the compensatory (asymptomatic) and decompensated stages [5]. Asymptomatic LC develops into the decompensated stage with the common clinical manifestations of ascites, hemorrhage, encephalopathy, and jaundice, conducing to decreased life quality and high mortality [6]. In China, approximately 3% of cases of compensatory LC (CLC) progress into decompensated LC annually and a 5-year death rate of 85% is estimated in patients with decompensated LC [7]. Despite liver transplantation can enhance survival rates, its clinical application is limited by donor shortage, high expenses, and severe post-transplantation complications [7]. Currently, liver biopsy is the major method available for clinical diagnosis of LC; however, it is not appropriate for all patients and 2–3% of patients may undergo multiple complications [8]. Early diagnosis and timely treatment are crucial for improving the outcomes of patients with LC. Therefore, there is an urgent need for the discovery of novel diagnostic markers for early-stage LC.

Increasing studies indicate that gut dysbiosis plays a vital role in the occurrence and development of LC [5, 9]. The gut microbiota is defined as the complicated aggregation of microbes in the gut, including bacteria, archaea, fungi, and viruses [10]. The gut microbiota produces a mass of metabolites that facilitate the interaction between the gut microbiota and the host [11]. Normal microbial composition acts as a natural defense barrier, which has significant implications for the acquisition of nutrients and regulation of the immune system and metabolic ability on the host [12]. The liver is a highly active region of metabolism and immune homeostasis, which impacts the microbiota by secreting and modulating various immunogenic molecules and metabolites into the gut [13]. The abnormities in the gut microbiota in LC patients are marked by the elevated abundance of latently pathogenic bacteria and descended amounts of advantageous bacteria [14]. Previous research observed a rise in *Streptococcaceae*, *Peptostreptococcaceae*, *Erysipelotrichaceae*, *Clostridiaceae*, and *Pasteurellaceae* and a reduction in *Acidaminococcaceae*, *Porphyromonadaceae*, *Prevotellaceae*, and *Bacteroidaceae in* the fecal samples from LC patients [14]. This suggests that alterations in the gut microbiota might serve as diagnostic signatures of LC, prompting us to investigate the status of the gut microbiota in CLC.

Microbiome analysis combined with metabolomics has been extensively applied as an effective method to figure out the correlation between health outcomes and microbiome [15]. Metabolomics is an assessment of multi-parametric metabolic reactions of multi-cellular systems, designed to determine and quantify different small molecules in multiple biological samples or specific physiological conditions [16]. As an emerging technique, metabolomics can not only be used to identify specific diagnostic biomarkers but also to clarify molecular mechanisms underlying specific pathology [17]. A previous study indicated that disordered gut microbiome conduced to LC progression, which might be associated with changed microbiome–metabolite interactions [8]. Hence, the integration of gut microbiota and metabolomics analyses is expected to obtain more efficient diagnosis biomarkers for CLC.

In this study, 16S ribosomal DNA (rDNA) sequencing and untargeted metabolomics were performed on feces and urine samples, respectively. The findings of our study revealed the differences in the gut microbiome and urine metabolites between CLC patients and normal controls. Besides, we constructed a diagnostic model for CLC by integrating gut microbiome with metabolomics analyses and verified the model. This study may provide novel early diagnostic biomarkers for LC and furnish a reference for further research into the pathogenesis of LC.

## Materials and Methods

### Human Subjects and Clinical Samples

Forty-one human individuals aged 40–50 were involved in the study, including 20 healthy volunteers (control) and 21 patients with CLC. These human individuals were enrolled in Tiantai People’s Hospital of Zhejiang Province (from 2018 to 2021). Patients were diagnosed with LC in terms of medical history, liver biopsy, clinical symptoms, laboratory tests, imaging tests, histological examinations, and complications. The diagnosis for CLC was based on the criteria described in Chinese guidelines on the management of liver cirrhosis (abbreviated version) [18]. No participants suffered hypertension, diabetes, inflammatory bowel disease, or necrotizing enteritis. Besides, none of these participants took proton pump inhibitors, antibiotics, or probiotics within 2 weeks before sample collection. Feces and urine samples were collected in the morning after an overnight fast, then delivered to the laboratory within 2 h on dry ice, and stored at -80℃ until further analyses. We performed the sample collection following the principle of voluntariness. Finally, 40 appropriate feces samples (control: 19; patient: 21) and urine samples (control: 20; patient: 20) were included for subsequent gut microbiota and urine metabolomics analyses, respectively. The sample sizes for these analyses conformed to a previous study [19]. The demographic and clinical data of subjects involved in this study are presented in Table S1. All participants signed written informed consent, and the experimental procedures were authorized by the Ethics Committee of Tiantai People’s Hospital of Zhejiang Province (TYLL2019-06). This study was in line with the Declaration of Helsinki.

### 16S rDNA Sequencing

According to the manufacturer’s instructions, the QIAamp DNA Mini Kit (Qiagen, Hilden, Germany) was used for DNA extraction from fecal samples. NanoDrop2000 spectrophotometer (Thermo Fisher Scientific, Waltham, MA, USA) was utilized to measure the concentration and purity of the DNA, and DNA quality was evaluated through 1% agarose gel electrophoresis. Then, the V3–V4 regions of bacterial 16S rDNA were amplified using the universal primers 338F, 5′-ACT CCT ACG GGA GGC AGC A-3′ and 806R, 5′-GGA CTA CHV GGG TWT CTA AT-3′. Following PCR amplification, the products were obtained by 2% agarose gel. The purified DNA fragments were then eluted using Tris–HCl and assessed using 2% agarose gel electrophoresis. The following conditions for amplification were applied: initial denaturation at 95 °C for 3 min, 30 cycles of denaturation at 95 °C for 30 s, annealing at 55 °C for 30 s, and primer elongation at 72 °C for 45 s. All amplicons were purified using Agencourt AMPure XP (Beckman Coulter, USA), which were quantified using Qubit dsDNA HS Assay Kit and Qubit 3.0 fluorometer (Thermo Fisher Scientific, Waltham, MA, USA). TruSeq DNA PCR-Free Sample Preparation Kit (Illumina, USA) was used for sequencing library generation and index code addition. The library construction process involved the following steps: (1) ligation of the “Y”-shaped adapter, (2) removal of self-ligated fragments using magnetic beads, (3) enrichment of the library through PCR amplification, and (4) denaturation of DNA using sodium hydroxide. Agilent 4200 Tapestation (Agilent Technologies, Palo Alto, CA, USA) was applied to determine library quality. Finally, the purified amplification fragment was incorporated into a library of PE 2 × 300 for sequencing on the HiSeq 2500 platform (Illumina, San Diego, CA, USA) following the manufacturer’s directions. Only high-quality reads were retained by removing all terminal bases with low quality (< Q20) using Trimmomatic (version 0.35).The sequence data related to this study have been deposited in the NCBI Short Read Archive (SRA) database (accession number: PRJNA1019460).

### Bioinformatics Analyses of 16S rDNA Sequencing

Ultra-fast FASTQ preprocessor fastp (version 0.19.4, https://github.com/OpenGene/fastp) [20] was used for quality control of raw sequencing data. For quality control, reads with a length of 300 bp were truncated at sites where the average quality score across a sliding window of 50 bp fell below 20 and truncated reads with a length of less than 50 bp were removed. Besides, the original sequences were compared using Burrows–Wheeler Aligner (BWA) software [21], and the contaminated reads with high similarity were removed, resulting in the optimized sequences. Double-ended splicing of original sequences was performed using fast length adjustment of short reads (FLASH; version 1.2.11, http://www.cbcb.umd.edu/software/flash) [22]. For splicing, the parameter of maximum mismatch rate between overlaps was limited to 0.2 and the length was longer than 10 bp. DADA2 (version 1.2.1, https://github.com/benjjneb/dada2) [23] was applied for denoising to identify amplicon sequence variants (ASVs). Taxonomy of the ASVs and species annotations (domain, kingdom, phylum, class, order, family, genus, and species) were performed using the naïve Bayesian classifier, Ribosomal Database Project (RDP; version 11.1, http://rdp.cme.msu.edu/) [24]. Clean tags were clustered into optimal taxonomic units (OTUs) with a similarity threshold of 97% using UPARSE (http://drive5.com/uparse/) [25]. The UCHIME (http://www.drive5.com/usearch/manual/uchime_algo.html) [26] was used to delete chimeric sequences. Then, a representative sequence was assigned to each OTU to obtain annotation information using the RDP classifier with the alignment threshold limited to 70% by comparison with SILVA database. The bar diagram, heatmap, and Circos diagram were drawn using “ggplot2,” “pheatmap,” and “RCircos” R packages to reflect the differences in microbial community composition between control and patient samples, respectively.

Pan/core-genome analyses of the 16S rDNA sequences were performed using “ggplot2” R package. Alpha diversity indicators, including sobs, ace, chao, shannon, simpson, shannoneven, simpsoneven, coverage, and Faith’s Phylogenetic Diversity (PD) indices, were calculated using QIIME software (version 1.9.1) [27]. Graphs of alpha diversity indexes, rarefaction curves (Sobs and Shannon indices), and rank-abundance curve were drawn via “ggplot2” R package. For beta diversity analysis, the QIIME software was used to calculate the unweighted UniFrac distance, followed by construction of the unweighted pair-group method with arithmetic mean (UPGMA) sample clustering tree using “ape” R package. Principal component analysis (PCA) was performed using “ropls” R package. Furthermore, principal coordinate analysis (PCoA) based on the UniFrac distance and nonmetric multidimensional scaling (NMDS) analysis were carried out using “vegan” R package.

### Urine Sample Preparation

First, 400-μL methanol (containing 1-μg/mL chlorophenylalanine as internal standard) was added to the urine sample (100 μL) in the 1.5-mL centrifuge tube. Then, the mixture was vortexed for 30 s and centrifuged for 10 min (14,000 × *g*; 4 °C), followed by vacuum drying. The supernatant (200 μL) was transferred to the autosamplers for subsequent ultra-performance liquid chromatography-mass spectrometry (UPLC-MS) analysis. Additionally, quality control (QC) samples were prepared by blending an equal portion of each urine sample. Prepared QC samples were inserted in every eight urine samples, which were subjected to regular analysis to evaluate the stability and repeatability of the instrumental experiment.

### Liquid Chromatography-Mass Spectrometry (LC–MS) Analysis

Sample separation for liquid chromatography analysis was performed using XSelect HSS T3 Column (2.1 mm × 100 mm; 1.8 µm; Waters, Milford, MA, USA). The mobile phase was composed of 0.1% formic acid in water (A) and 0.1% formic acid in acetonitrile (B). The conditions of gradient elution were applied as follows: 0–3.5 min, 0–24.5% B, 0.4 mL/min (flow rate); 3.5–5 min, 24.5%-65% B, 0.4 mL/min; 5–5.5 min, 65–100% B; 5.5–7.4 min, 100%B, 0.4–0.6 mL/min; 7.4–7.6 min; 100–51.5%B, 0.6 mL/min; 7.6–7.8 min; 51.5–0%B, 0.6–0.5 mL/min; 7.8–9 min; 0%B, 0.5–0.4 mL/min; 9–10 min; and 0%B, 0.4 mL/min. Other parameters of the analysis were as follows: injection volume, 2 μL; column temperature, 40 °C; and sample room temperature, 10 °C.

For mass spectrometry analysis, positive- and negative-ion modes were applied using the electrospray ionization source of the UHPLC-Q Exactive HF-XQ system (Thermo Fisher). The mass scan range was set to 50–1000 m/z. The conditions of electrospray ionization were as follows: sheath gas flow rate, 50 arbitrary units; auxiliary gas flow rate, 13 arbitrary units; heater temperature, 425 °C; capillary temperature, 325 °C; ion spray voltage floating, 3500 V (positive mode) and −3500 V (negative mode); collision energy, 20/40/60 eV; full MS resolution, 60,000; and MS/MS resolution, 7500.

### Urine Metabolomics Analyses

Raw data were entered into the Progenesis QI software (version 2.2, Waters) for data pre-processing, including baseline filtering, peak identification, alignment, integration, retention time correction, and normalization. Then, a data matrix composed of retention time, mass-to-charge ratio values, and peak intensity was obtained. Metabolite annotation was performed using Human Metabolome Database (HMDB; version 5.0, http://www.hmdb.ca/) [28], Kyoto Encyclopedia of Genes and Genomes (KEGG; www.genome.jp/kegg) [29], and Lipidmaps database (http://www.lipidmaps.org/) [30]. Data analysis was conducted using the online platform Majorbio Cloud (https://cloud.majorbio.com). Metabolomics features presenting more than 20% of missing values were eliminated from the analysis. After normalization, variables with relative standard deviation > 30% of QC samples were removed. The final data matrix was obtained after log10 transformation and used for subsequent analysis.

PCA and orthogonal partial least-squares discrimination analysis (OPLS-DA) were carried out to determine the distinctions among variables using “ropls” R package. The reliability of OPLS-DA was evaluated via five-fold cross-validation and permutation test with the criteria “R^2^Y (goodness of fit indicator) and Q^2^ (predictive capacity indicator) > 0.5” [31]. On the basis of OPLS-DA analysis, metabolites meeting the criterion of “variable importance in projection (VIP) > 1” were selected as key variables. The following criteria were applied to identify differentially expressed metabolites: VIP > 1; *p* < 0.05 [32]. The top 50 differential metabolites were screened based on the values of |log_2_ fold change| from high to low. The heatmap reflecting the distribution of these metabolites in each sample was drawn by “pheatmap” R package. Besides, Pearson correlation analysis was conducted to assess the links among the top 50 differential metabolites, which was visualized by “corrplot” R package. Then, all the differential metabolites were subjected to pathway enrichment analysis using MetaboAnalyst (version 4.0, https://www.metaboanalyst.ca) [33], followed by network analysis using Metscape [34].

### Construction and Verification of Diagnostic Model for CLC

Data of gut microbiota and metabolomics analyses were merged and a multi-factor classification model was built based on the least absolute shrinkage and selection operator (LASSO) regression algorithm using “caret” R package (https://github.com/topepo/caret/) [35]. Then, the merged gut microbiota and metabolomics data were randomly divided into training (29 samples) and testing (10 samples) sets to avoid overfitting of the model. Through five-fold cross-validation, the optimal diagnostic markers were identified according to the lambda. min value. The final diagnostic model was generated, and the area under the curve (AUC) of the receiver operating characteristic (ROC) was calculated using SPSS software (version 26.0, IBM, Armonk, NY, USA) and visualized using “pROC” R package. Otherwise, Pearson correlation analysis was applied to clarify the relations among the diagnostic markers and visualized by “corrplot” R package.

### Statistical Analysis

Statistical analyses were undertaken using SPSS software (version 26.0, IBM) and R software (version 4.2.2). Data are exhibited as means ± standard deviation (SD) or medians with ranges. Fisher’s test was applied to identify significantly enriched pathways related to differential metabolites. Student’s t test was utilized for comparisons of the differences between two groups. The criterion for statistical significance is *p* < 0.05. For controlling multiple hypothesis testing, we employed Bonferroni correction method to adjust statistical significance.

## Results

### 16S rDNA Sequencing Data Analyses

In this study, 16S rDNA sequencing was initially performed on 40 feces samples, including 19 from healthy volunteers (control) and 21 from patients with CLC, resulting in 2,910,360 raw sequences. Quality control was performed using fastp with the following criteria: reads with a length of 300 bp were truncated at sites where the average quality score across a sliding window of 50 bp fell below 20; truncated reads with a length of less than 50 bp were deleted. Furthermore, The raw sequences were compared using BWA software [21], and the contaminated reads with high similarity were deleted, generating the optimized sequences. In this study, a total of 2,362,245 high-quality sequences with the mean length of 412.57 bp were obtained (Table S2).

Pan- and core-genome analyses were utilized to determine whether the current sequencing sample size is sufficient by evaluating alterations in total, and core ASVs as the sample number were elevated. Pan-genome analysis revealed that the total number of ASVs tended to be maximized with the increasing number of samples (Figure S1A). Moreover, core-genome analysis showed that the number of key ASVs descended and then remained unchanged with the growing number of samples (Figure S1B). Furthermore, the rank-abundance curves tended to be smooth, demonstrating that most of the microbial diversity on genus level in each sample had already been acquired at the present sequencing depth (Figure S1C).

### Alpha and Beta Diversity Analyses

**Fig. 1:**
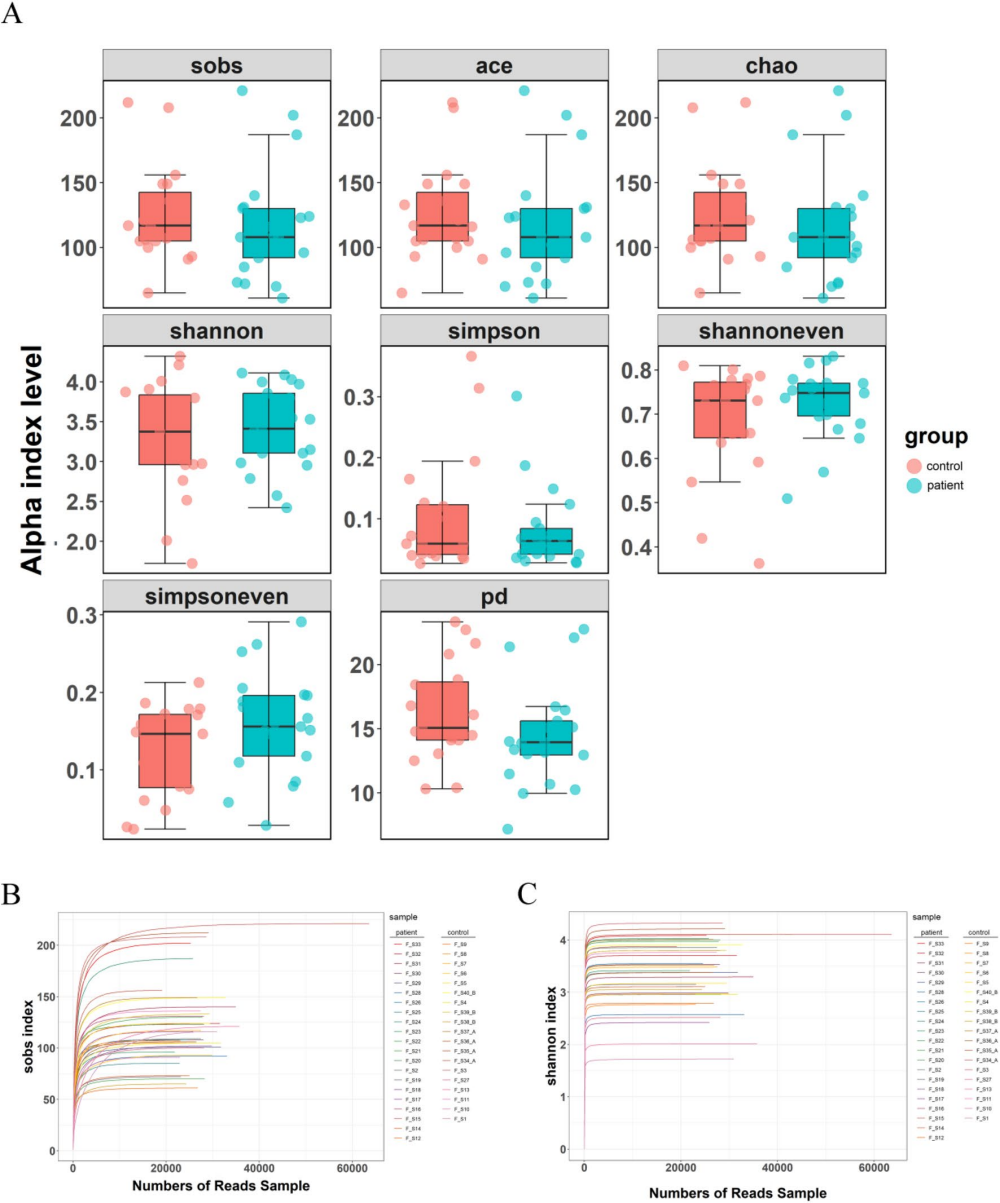
Changed alpha diversity of gut microbiota in patients with CLC. **A** Box and whisker plots of alpha diversity indexes, including sobs, ace, chao, shannon, simpson, shannoneven, simpsoneven, and PD of control and patient samples. **B** Rarefaction curve of sobs index. **C** Rarefaction curve of shannon index. Alpha diversity indices were calculated using QIIME software (version 1.9.1) and “ggplot2” R package was used for visualization. Control group, *n* = 19; patient group, *n* = 21. CLC, compensatory liver cirrhosis; PD, Faith’s Phylogenetic Diversity

Based on the ASVs, alpha diversity analyses were performed to evaluate the richness and diversity. In this study, we assessed 9 alpha diversity indicators, including Sobs, ace, chao, shannon, simpson, shannoneven, simpsoneven, coverage, and PD indexes. Sobs, ace, and chao indices reflect community richness [36]; shannon, simpson, and PD reflect community diversity [36, 37]; shannoneven and simpsoneven reflect community evenness [37]; and coverage index reflects community coverage [38]. Our data showed that sobs, ace, chao, simpson, and PD indexes of patient samples were lower than those of control samples, while simpsoneven index was higher in the patient samples (Fig. 1A). These alpha diversity indices of each sample are listed in Table S3. Furthermore, rarefaction curves were built using alpha diversity indices (Sobs and Shannon) for each sample to reflect the microbial diversity of each sample at different sequencing amounts. Here, the rarefaction curves tended to be flat, indicating that the amount of present sequencing data was sound and large enough to mirror the majority of microbial information in the feces samples (Fig. 1B, C).

**Fig. 2:**
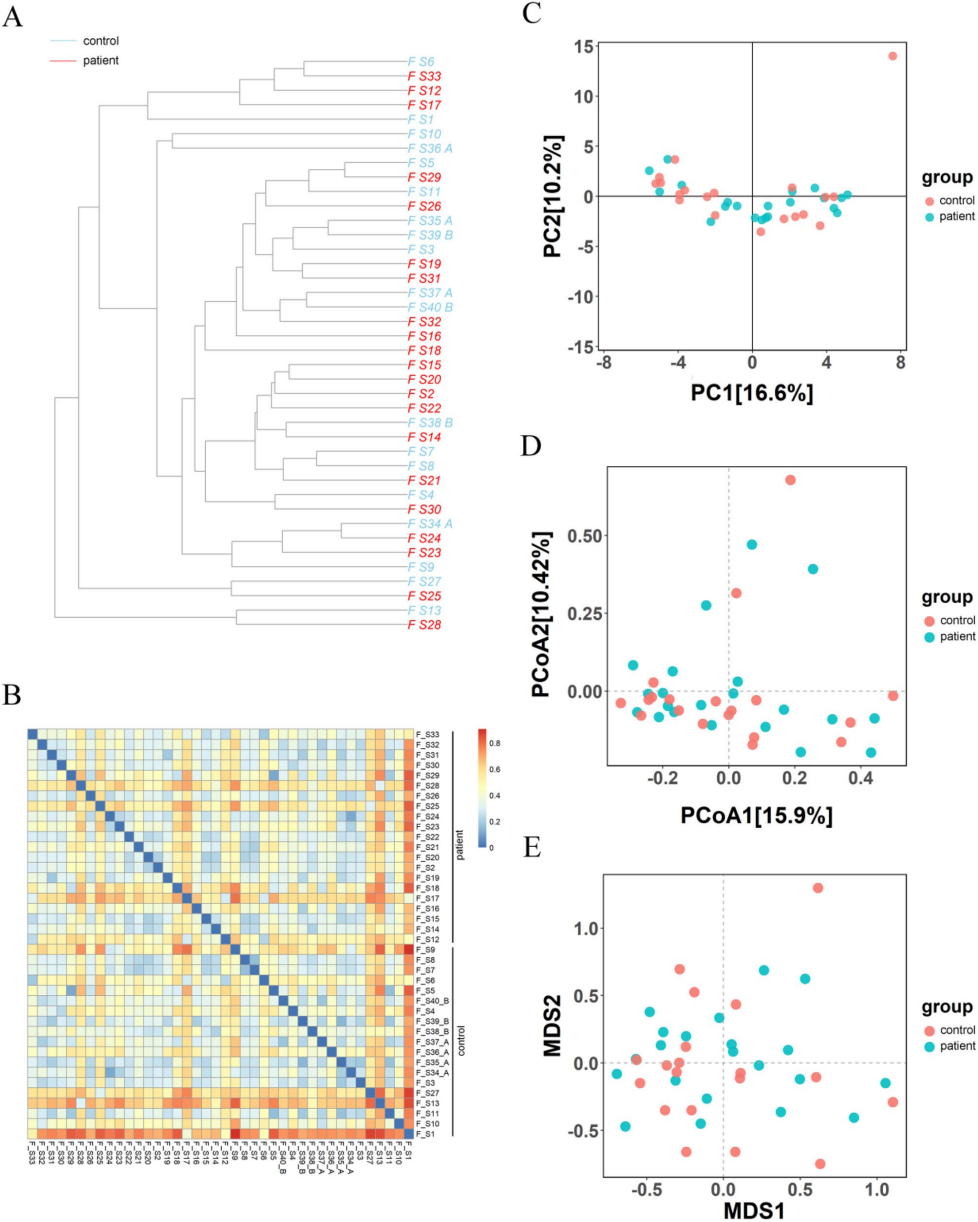
Altered beta diversity of gut microbiota in patients with CLC. **A** UPGMA clustering tree of the control and patient samples. The unweighted UniFrac distance was calculated using QIIME software (version 1.9.1) and visualized by “ape” R package. **B** Heatmap of the unweighted UniFrac distance among the control and patient samples, visualized by “pheatmap” R package. **C** PCA analysis of control and patient samples using “ropls” R package. **D** PCoA analysis of control and patient samples using “vegan” R package. **E** NMDS analysis of control and patient samples using “vegan” R package. Control group, *n* = 19; patient group, *n* = 21. CLC, compensatory liver cirrhosis; UPGMA, unweighted pair-group method with arithmetic mean; PCA, principal component analysis; PCoA, principal coordinate analysis; NMDS, nonmetric multidimensional scaling

Beta diversity analyses were conducted to figure out the similarity or difference in the community composition between controls and patients. UPGMA clustering tree analysis is a data visualization approach to reflect the degree of variation in microbial evolution in multiple samples [39]. UPGMA clustering tree analysis and related heatmap showed different degrees of discrepancies and similarities in the microbial composition among the samples, as the distances varied between every two samples (Fig. 2A, B). PCA reflects the discrepancies in various datasets on a two-dimensional coordinate plot through variance decomposition. The higher the similarity in the sample composition between two samples, the shorter the distance between them presented in the PCA plot [40]. According to PCA on ASV level, the vast majority of dots (samples) in patient group were separated from those in control group (PC1 = 16.6%; PC2 = 10.2%; Fig. 2C). PCoA is a data dimensionality reduction analysis method, allowing visualization of discrepancies between samples [41]. In this study, most of the dots in patient group were significantly isolated from those in control group in the pots of PCoA (PCoA1 = 15.9%; PCoA2 = 10.42%; Fig. 2D). NMDS is also a widely used visualization method to study sample differences [42]. NMDS on ASV level further demonstrated the differences in the overall microbial composition between normal subjects and CLC patients (Fig. 2E).

### Microbial Community Structure Analyses

**Fig. 3:**
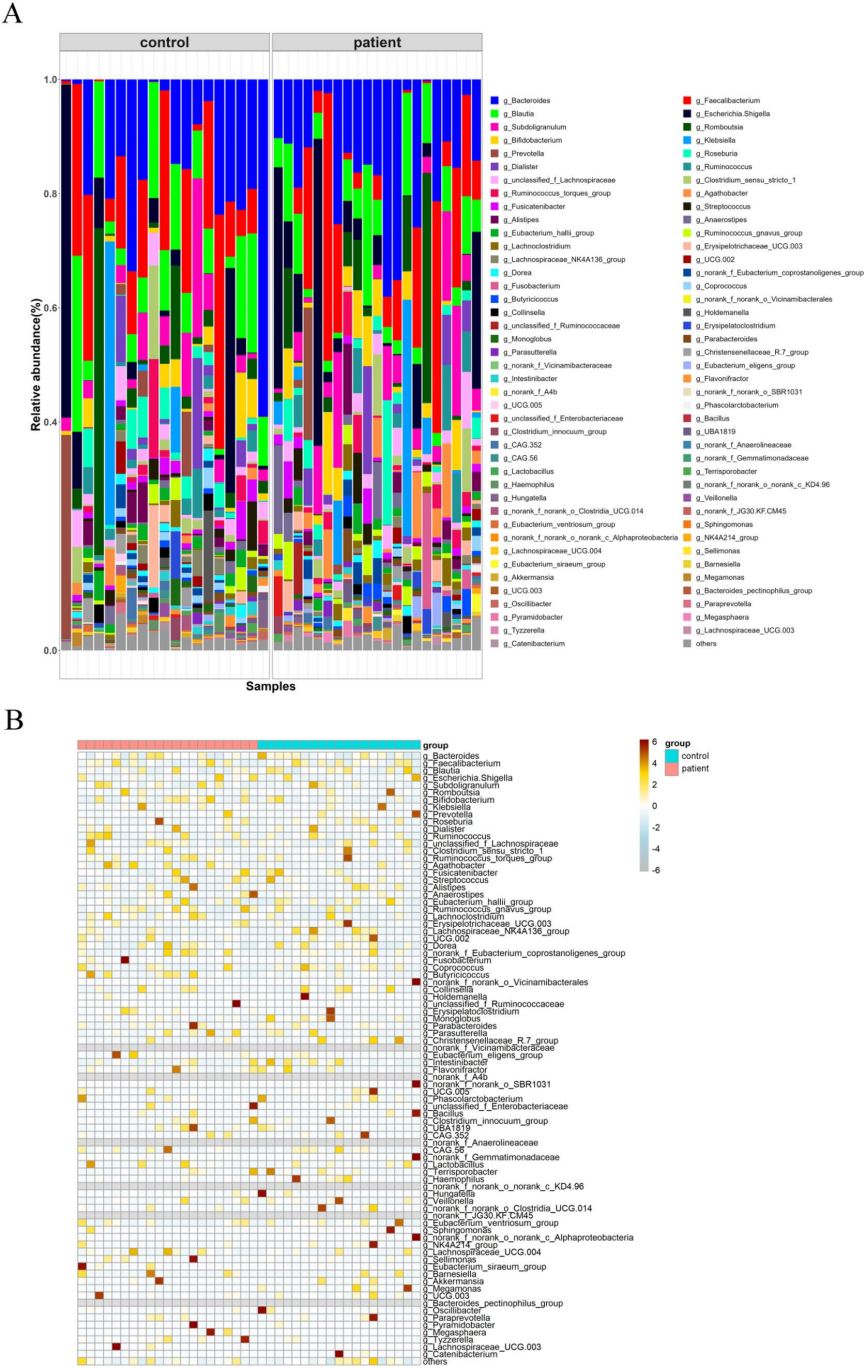
Changed microbial community composition in patients with CLC. **A** Bar diagram of the relative abundance of different microbial taxa on genus level of each control and patient sample, visualized by “ggplot2” R package. **B** Heatmap of the microbial community composition of each control and patient sample, visualized by “pheatmap” R package. Control group, *n* = 19; patient group, *n* = 21. CLC, compensatory liver cirrhosis

**Fig. 4:**
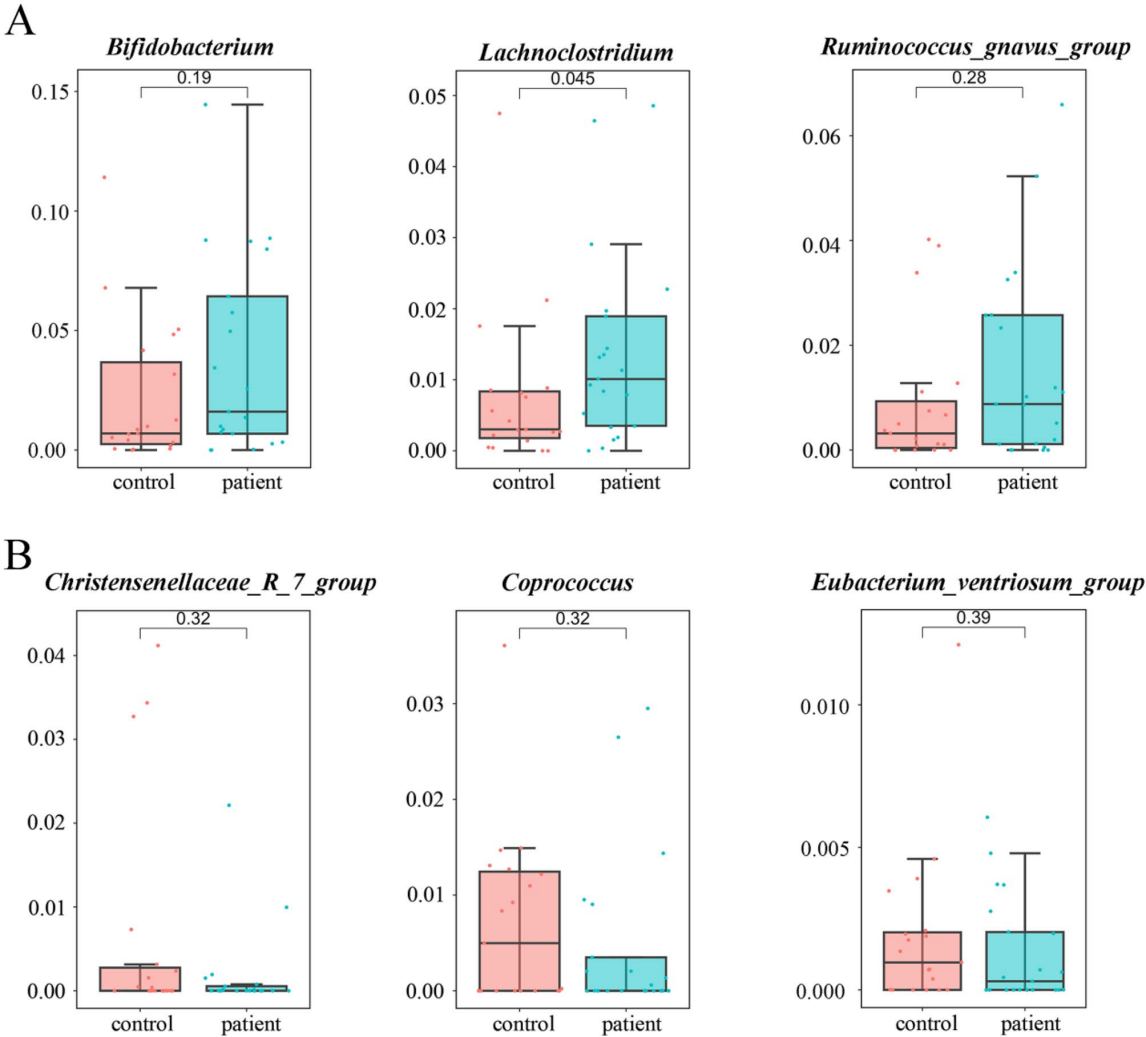
Altered microbial community composition in patients with CLC. **A** Box and whisker plots of elevated average abundance of microbial taxa in patient samples compared with that in control samples. *Bifidobacterium*, *Lachnoclostridium*, and *Ruminococcus_gnavus_group* were displayed. **B** Box and whisker plots of reduced average abundance of microbial taxa in patient samples compared with that in control samples. Box and whisker plots were visualized by “ggplot2” R package. Control group, *n* = 19; patient group, *n* = 21. *Christensenellaceae_R_7_group*, *Coprococcu*s, and *Eubacterium_ventriosum_group* were displayed. CLC, compensatory liver cirrhosis

The bar diagram, heatmap, Circos diagram, and histogram of microbial average abundance were used to clarify the discrepancies in microbial community structure between control and patient fecal samples. The bar diagram showed variations in the relative abundance of different microbiota among these 40 fecal samples on the genus level (Fig. 3A). *Bacteroides* (a maximum of 59% in control, 38% in patient), *Faecalibacterium* (a maximum of 41% in control, 49% in patient), *Blautia* (a maximum of 31% in control, 24% in patient), and *Escherichia*. *Shigella* (a maximum of 58% in control, 51% in patient) were the major genera in these samples (Table S4). Besides, the heatmap illustrated that each genus was differently distributed in each sample, which further verified the findings of the bar diagram analysis (Fig. 3B). The Circos diagram directly reflected the distribution of each genus in control and patient samples. In the Circos diagram, the overall abundance of some genera (e.g., *Blautia*) in patient samples was lower than that of control samples, while some genera (e.g., *Escherichia*. *Shigella*) showed higher overall abundance in patient samples (Figure S2). Through analysis of average microbial abundance, we found changed microbial abundance of some genera in patient samples compared with that of control samples. For instance, the average abundance of *Bifidobacterium*, *Lachnoclostridium*, and *Ruminococcus_gnavus_group* was elevated in patient samples compared with that in control samples (Fig. 4A). However, decreased average abundance of *Christensenellaceae_R_7_group*, *Coprococcu*s, and *Eubacterium_ventriosum_group* was observed in patient samples by comparison with control samples (Fig. 4B).

### Urine Metabolomics Analysis

Metabolomics analysis was conducted on 40 urine samples (control: 20; patient: 20). Through UPLC-MS and data pre-processing, 9,351 peaks were picked and 2,637 metabolites with specific names were identified (Table S5).

**Fig. 5:**
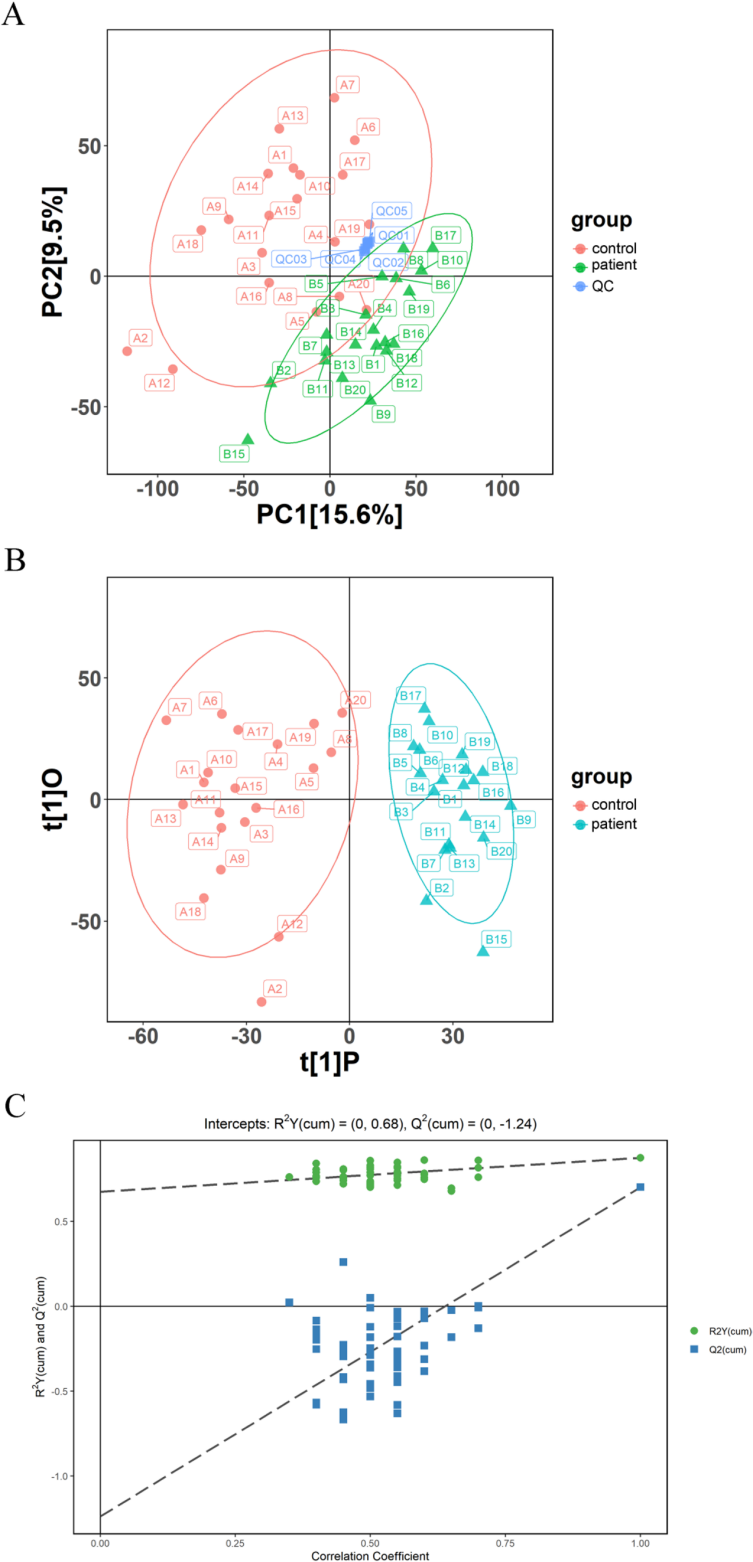
Altered urine metabolome in patients with CLC. **A** PCA analysis of urine samples in control and patient groups based on QC samples using “ggplot2” R package. Control group, *n* = 20; patient group, *n* = 20; QC, n = 5. **B** OPLS-DA analysis of urine samples in control and patient groups using “ropls” R package. Control group, *n* = 20; patient group, *n* = 20. **C** OPLS-DA permutation test in the negative and positive ion modes. CLC, compensatory liver cirrhosis; PCA, principal component analysis; QC, quality control; OPLS-DA, orthogonal partial least-squares discrimination analysis

To assess the metabolic variations in CLC patients compared with healthy individuals, PCA and OPLS-DA analyses were conducted on the patient and control urine samples. According to PCA analysis based on QC and testing samples, PC1 and PC2 accounted for 15.6% and 9.5% variables, respectively; significantly, the majority of patient samples were isolated from control samples (Fig. 5A). Besides, a notable separation tendency was observed between control and patient samples based on the OPLS-DA model (Fig. 5B). Further, the OPLS-DA model exhibited a favorable explanatory ability (*R*^2^*Y* = 0.876; *p* value < 0.01) and predictive capacity (*Q*^2^ = 0.703, *p* value < 0.01) through five-fold cross-validation and permutation test (Fig. 5C).

### Determination of Differential Metabolites

**Fig. 6:**
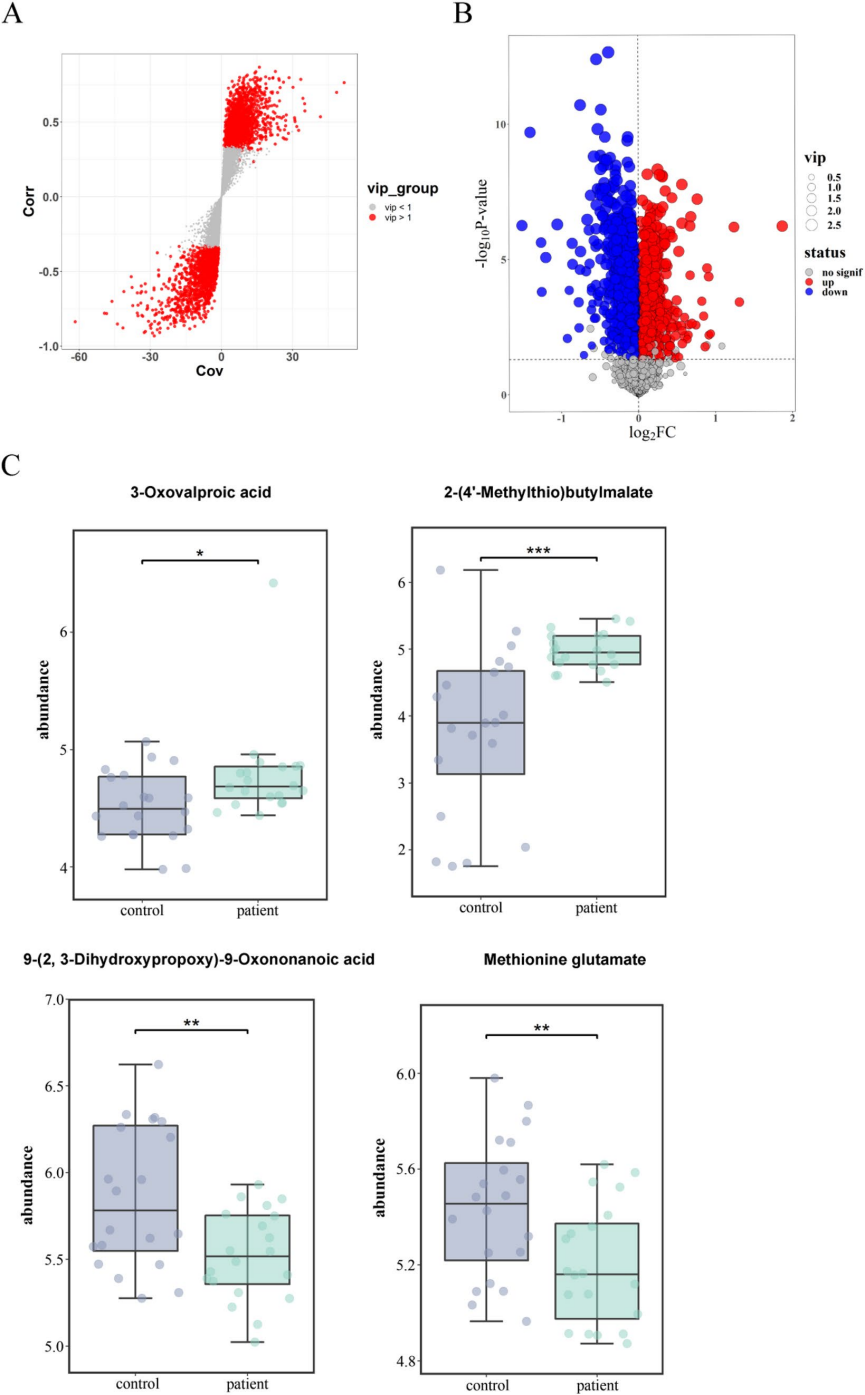
Identification of differential metabolites in patients with CLC. **A** Plot of VIP scores of the identified metabolites. Red dots denote the metabolites with VIP > 1; gray dots denote the metabolites with VIP < 1. **B** Volcano plot of differential metabolites. Red dots denote upregulated differential metabolites; blue dots denote downregulated differential metabolites. VIP > 1; *p* < 0.05. **C** Box and whisker plots of the abundance of differential metabolites in control and patient samples. “ggplot2” R package was used for visualization. Control group, *n* = 20; patient group, *n* = 20. 3-Oxovalproic acid, 2-(′-methylthio)butylmalate, 9-(2, 3-dihydroxypropoxy)-9-oxononanoic acid, and methionine glutamate were displayed. ^*^p < 0.05, ^**^p < 0.01, and ^***^p < 0.001 vs control group. CLC, compensatory liver cirrhosis; VIP, variable importance in projection

In the plot of VIP scores of the identified metabolites, the red dots denoted the metabolites with VIP > 1 and were regarded as potential biomarkers for CLC based on the OPLS-DA model (Fig. 6A). Notably, 841 differential metabolites were identified under the criteria of “VIP > 1; *p* < 0.05” and Bonferroni correction was used to adjust the *p* values (Table S6). The volcano plot showed 533 upregulated metabolites (red dots) and 308 downregulated metabolites (blue dots) (Fig. 6B). For example, the abundance of 3-Oxovalproic acid and 2-(4′-Methylthio)butylmalate was markedly upregulated in patient samples compared with those in control samples (*p* < 0.05; Fig. 6C). Conversely, 9-(2, 3-Dihydroxypropoxy)-9-Oxononanoic acid and methionine glutamate exhibited notable downregulated abundance in patient samples by comparison with those in control samples (*p* < 0.01; Fig. 6C). The distribution of the top 50 differential metabolites in all samples was displayed in the heatmap (Figure S3A).

### Correlation and Pathway Analyses of Differential Metabolites

**Fig. 7:**
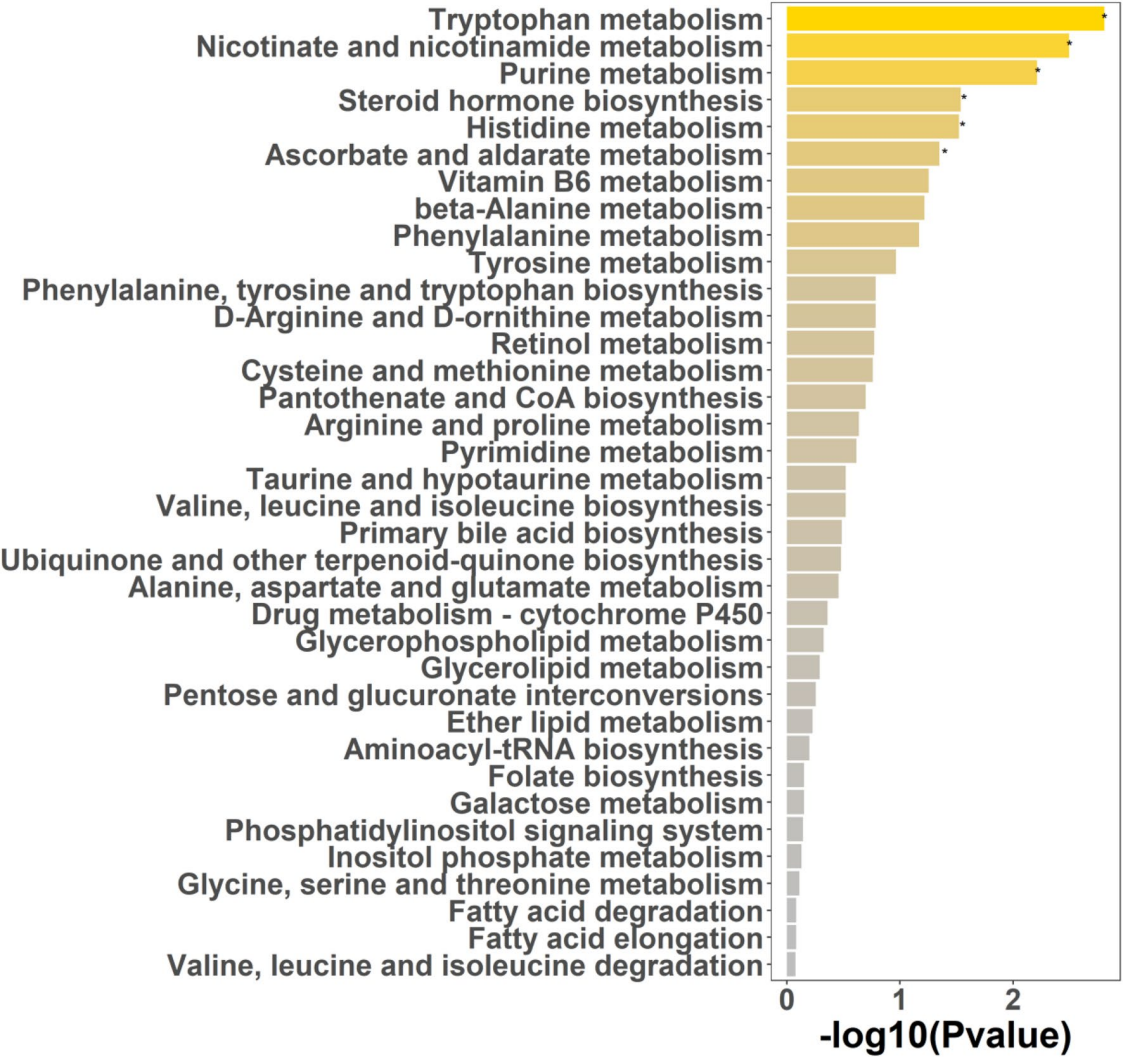
Pathway enrichment analysis of differential metabolites in CLC patients using MetaboAnalyst software. CLC, compensatory liver cirrhosis

Also, Pearson correlation analysis was performed to figure out the correlations among these 841 differential metabolites. Results showed multiple relations among the top 50 differential metabolites (Figure S3B). Pathway enrichment analysis showed that the differential metabolites were concentrated in 36 pathways. The top 5 significantly enriched pathways included tryptophan metabolism, nicotinate and nicotinamide metabolism, purine metabolism, steroid hormone biosynthesis, and histidine metabolism (*p* < 0.05; Fig. 7). The corresponding networks of the top 5 pathways are displayed in Figure S4.

### Establishment and Validation of a Diagnostic Model for CLC

**Fig. 8:**
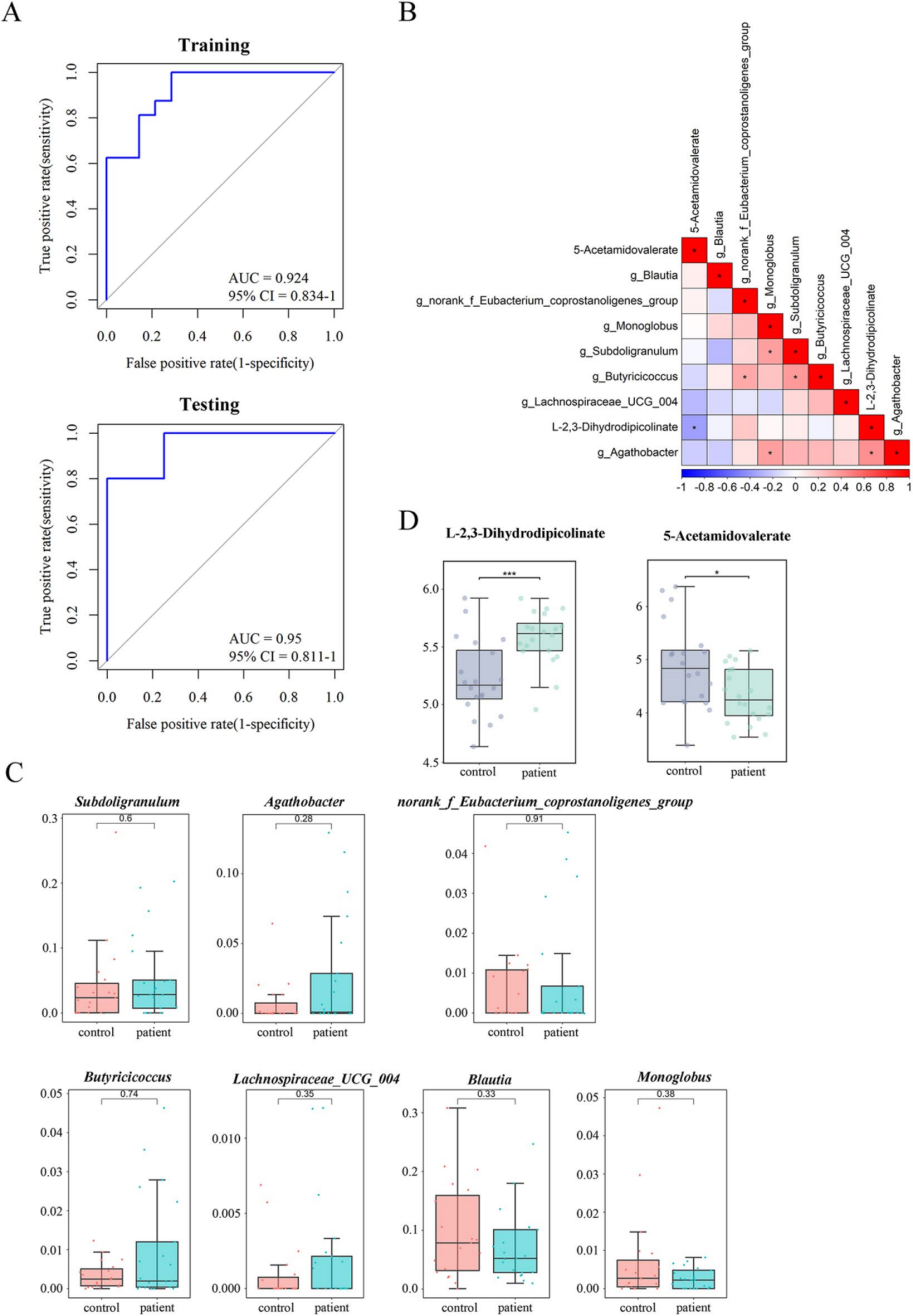
Construction and validation of the diagnostic model for CLC. **A** ROC analysis in the training and testing sets using “pROC” R package. **B** Heatmap of correlation analysis of the 9 diagnostic biomarkers for CLC, visualized by “corrplot” R package. **C** Box and whisker plots of the average abundance of the 7 gut microbiota biomarkers in control and patient samples, visualized by “ggplot2” R package. Control group, *n* = 19; patient group, *n* = 21. **D** Box and whisker plots of the abundance of the 2 metabolite biomarkers in control and patient samples. Control group, *n* = 20; patient group, *n* = 20. ^*^p < 0.05 and ^***^p < 0.001 vs control group. CLC, compensatory liver cirrhosis; ROC, receiver operating characteristic

Through five-fold cross-validation, a 9-marker diagnostic model for CLC was built. This model consisted of 7 intestinal microorganisms (*Blautia*, *Subdoligranulum*, *Agathobacter, norank_f_Eubacterium_coprostanoligenes_group*, *Butyricicoccus*, *Monoglobus*, and *Lachnospiraceae_UCG_004*) and 2 metabolites (L-2,3-Dihydrodipicolinate and 5-Acetamidovalerate) (Table 1). Notably, ROC curves showed that the 9-marker model had optimal performance with favorable AUC values in both training set (AUC = 0.924, 95% CI = 0.834–1) and testing set (AUC = 0.95, 95% CI = 0.811–1) (specificity > 90%, sensitivity > 90%; Fig. 8A). Furthermore, the heatmap showed various relations among these intestinal florae and metabolites (Fig. 8B). For example, *Agathobacter* was positively related to L-2,3-Dihydrodipicolinate (*r* = 0.42), while negatively related to 5-Acetamidovalerate (*r* = 0.18). *Norank_f_Eubacterium_coprostanoligenes_group* was positively correlated with L-2,3-Dihydrodipicolinate (*r* = 0.2) and 5-Acetamidovalerate (*r* = 0.05). In this model, the relative abundance of *Subdoligranulum*, *Agathobacter*, *norank_f_Eubacterium_coprostanoligenes_group*, *Butyricicoccus*, and *Lachnospiraceae_UCG_004* was increased in CLC patients, whereas *Blautia* and *Monoglobus* were decreased (Fig. 8C). Otherwise, the abundance of L-2,3-Dihydrodipicolinate was markedly elevated in CLC patients, while 5-Acetamidovalerate was reduced (*p* < 0.05; Fig. 8D**)**.

**Table 1:** Parameters of the optimal diagnostic model for CLC

	Marker	Coef
(Intercept)		0.095743656
g_Blautia	g_Blautia	−3.111189964
g_Subdoligranulum	g_Subdoligranulum	1.135761231
g_Agathobacter	g_Agathobacter	1.495834703
g_norank_f_Eubacterium_coprostanoligenes_group	g_norank_f_Eubacterium_coprostanoligenes_group	0.750636129
g_Butyricicoccus	g_Butyricicoccus	5.608887244
g_Monoglobus	g_Monoglobus	−2.592074156
g_Lachnospiraceae_UCG_004	g_Lachnospiraceae_UCG_004	2.253676077
metab_403	L-2,3-Dihydrodipicolinate	0.136301022
metab_7496	5-Acetamidovalerate	–0.131219474

## Discussion

LC is a primary cause of death and a common outcome of multiple progressive liver disorders, leading to more than one million deaths worldwide each year [43, 44]. In the present study, 16S ribosomal DNA (rDNA) sequencing and untargeted metabolomics were used to investigate the status of the gut microbiota and metabolome in patients with CLC. We observed differences in the intestinal microorganisms and urinary metabolites between healthy participants and patients with CLC. Finally, a 9-signature diagnostic model for CLC based on the differential gut and metabolites was constructed and verified to be reliable. The diagnostic model comprised 7 intestinal microorganisms (*Blautia*, *Subdoligranulum*, *Agathobacter*, *norank_f_Eubacterium_coprostanoligenes_group*, *Butyricicoccus*, *Monoglobus*, and *Lachnospiraceae_UCG_004*) and 2 metabolites (L-2,3-Dihydrodipicolinate and 5-Acetamidovalerate).

Accumulating evidence indicates that disordered gut microbiome is implicated in the pathogenesis of LC [5, 9]. Currently, impaired gut-liver axis has been recognized as perturbed intestinal microbial structure, disrupted intestinal barrier, and enhanced permeability of intestinal barrier and found in various liver disorders [10, 45]. In this study, we found that the microbial alpha diversity indexes (sobs, ace, chao, simpson, and PD) were decreased in patients with CLC compared with those in healthy participants. Moreover, the beta diversity analysis showed that the microbial composition in patients with CLC significantly varied from that in healthy participants. These results suggest that abnormalities in gut microbiome occurred in patients with CLC, which may conduce to the onset of LC. We found elevated abundance of the key microorganisms, *Subdoligranulum*, *Agathobacter*, *norank_f_Eubacterium_coprostanoligenes_group*, *Butyricicoccus*, and *Lachnospiraceae_UCG_004* in patients with CLC compared with those in healthy participants. Oppositely, the abundance of *Blautia* and *Monoglobus* was reduced in patients with CLC.

*Subdoligranulum*, *Butyricicoccus*, and *Agathobacter* are the common producers of butyrate, a short-chain fatty acid (SCFA) [46, 47]. SCFAs, including acetate, propionate, and butyrate, are the major energy source for colonocytes, which have been proven to enhance the function of gut barrier [48, 49]. The liver is exposed to inflammatory signaling by impaired gut barrier, resulting in liver injury [49]. Usually, SCFAs are thought to be advantageous to the body. However, a previous study indicated that increased SCFAs could enhance liver inflammation and fibrosis, conducing to the progression of hepatocellular carcinoma [50]. It was previously revealed that increased *Agathobacter* induced by protein intake could promote the development of hepatic encephalopathy in LC [51]. In terms of *norank_f_Eubacterium_coprostanoligenes_group*, little is known about its role in LC development. Nevertheless, a recent study showed that *norank_f_Eubacterium_coprostanoligenes_group* level was notably elevated in rheumatoid arthritis (RA) [52]. Further, it was revealed that lowering *norank_f_Eubacterium_coprostanoligenes_group* and the overall level of SCFAs could alleviate RA [52]. Thus, we can hypothesize the crosstalk between *norank_f_Eubacterium_coprostanoligenes_group* and SCFAs in the inflammatory mechanism in LC. *Lachnospiraceae* species are also involved in the production of butyrate. Previous data observed a rise in *Lachnospiraceae_UCG_004* in human immunodeficiency virus (HIV) patients [53]. Moreover, HIV infection has been reported to contribute to LC [54]. *Blautia* are anaerobic bacteria with a probiotic feature, extensively found in mammalian feces and guts and they can produce acetate [55, 56]. Lately, a reduction in the abundance of *Blautia* has been found in patients with liver injury [57]. *Monoglobus* have been demonstrated to be implicated in dietary fiber fermentation and related to healthy communities [58]. A recent study has indicated that *Monoglobus* are specialized pectin-degrading bacteria linked with neutrophilic inflammation and serious liver damage [59]. Downregulation of *Monoglobus* promotes systemic inflammation, as evidenced by recent studies [59, 60]. Taken together, these findings suggest that these potential bacteria markers may participate in the pathogenesis of early-stage LC by regulating SCFA production and inflammatory responses.

Increasing data indicate that aberrant gut microbiome is closely associated with metabolic alterations in LC development [8, 11]. Abnormal gut-liver axis results in the transposition of microorganisms and their products, such as lipopolysaccharide and other metabolites into the portal bloodstream, straightly directed at the liver [45]. Through PCA and OPLS-DA analyses, we observed notable differences in the urine metabolome between CLC patients and healthy subjects. Specifically, we obtained 841 differential metabolites in CLC patients, including 533 metabolites upregulated and 308 downregulated.

Through pathway enrichment analysis, we observed that these differential metabolites were primarily concentrated in pathways, such as tryptophan metabolism, purine metabolism, and steroid hormone biosynthesis. Reduced tryptophan, elevated tryptophan-associated enzymes, and increased downstream metabolites are related to aggravated metabolic inflammation and fibrosis [45]. The tryptophan catabolic responses are mediated by intestinal bacteria, such as *Ruminococcus gnavus* [61]. A previous study demonstrated tryptophan metabolism was impaired in LC patients, which might be implicated in the pathogenesis of LC [62]. Purine metabolism acts as a switch in various biological processes (e.g., energy generation and DNA/RNA synthesis) [63]. Abnormal purine metabolism can conduce to the development of multiple disorders, especially hyperuricemia [64]. Lately, notable alterations in purine metabolic pathways have been observed in LC mice, indicating that dysregulated purine metabolism might give rise to LC [11]. Steroid hormones modulate a variety of biological mechanisms, principally in the reproductive system and multiple metabolic pathways [65]. As the pivotal metabolic organ, the liver acts as a key part in the homeostasis of steroid hormones [65]. Aberrant steroid hormone levels have been demonstrated to be closely linked with several liver conditions. For example, hypoestrogenism can result in the occurrence of and progression of NAFLD in post-menopausal females [66]. Otherwise, a previous study revealed that lacking of the major androgen, testosterone, might lead to sarcopenia in male patients with LC [67]. To sum up, these data indicate the involvement of various disturbed metabolic pathways in the onset and development of LC.

In terms of the metabolite markers, the abundance of L-2,3-Dihydrodipicolinate was increased in CLC patients, while 5-Acetamidovalerate was decreased. To our knowledge, L-2,3-Dihydrodipicolinate is a member of the class of alpha amino acids and derivatives and a major metabolite, found in all living organisms, from bacteria to humans. However, few investigations regarding l-2,3-Dihydrodipicolinate have been carried out. Otherwise, 5-Acetamidovalerate belongs to the straight chain fatty acid family, which can be formed by the enzymatic reduction of 5-aminopentanoate or enzymatic oxidation of 2-keto-6-acetamidocaproate (http://www.hmdb.ca/). Accumulating evidence indicates that disrupted fatty acid levels conduce to liver diseases, including LC [68, 69]. A previous investigation found that altered 5-Acetamidovalerate was associated with inflammation in aging mice, and it was positively related to *Ruminococcus* [70]*.* These results suggest that the onset and development of LC may be attributed to the abnormal metabolism of alpha amino acids and straight chain fatty acids.

Nonetheless, this study has some limitations need to be paid attention to. First, a larger number of human subjects are required to validate the sequencing and metabolomics analyses. Second, the crosstalk between microbiome and metabolome in CLC should be thoroughly analyzed. Third, the potential diagnostic model for CLC remains to be clinically confirmed.

## Conclusion

In this study, we determined a 9-signature diagnostic model for CLC, including *Blautia*, *Subdoligranulum*, *Agathobacter, norank_f_Eubacterium_coprostanoligenes_group*, *Butyricicoccus*, *Monoglobus*, *Lachnospiraceae_UCG_004*, L-2,3-Dihydrodipicolinate, and 5-Acetamidovalerate. Besides, abnormal tryptophan, nicotinate and nicotinamide, purine, and histidine metabolism, and steroid hormone biosynthesis were implicated in the pathogenesis of CLC. This study may provide a novel strategy for early diagnosis and treatment of LC and a basis for the discovery of drug targeting microbiome or metabolism for CLC.

## Supplementary Information

Below is the link to the electronic supplementary material.

Supplementary file1 Figure S1. Analyses of 16S rDNA sequencing data. (A) Pan-genome analysis of 16S rDNA sequencing on ASV level. (B) Core-genome analysis of 16S rDNA sequencing on ASV level. (C) Rank-abundance curve on genus level. Analyses were performed using “ggplot2” R package. Control group, n = 19; patient group, n = 21. rDNA, ribosomal DNA; ASV, amplicon sequence variant (TIF 8037 KB)

Supplementary file2 Figure S2. Circos diagram of the microbial community composition of each control and patient sample using “RCircos” R package. Control group, n = 19; patient group, n = 21 (TIF 6067 KB)

Supplementary file3 Figure S3. Analyses of the top 50 differential metabolites. (A) Heatmap of the distribution of the top 50 differential metabolites in each control and patient sample, visualized by “pheatmap” R package. Control group, n = 20; patient group, n = 20. (B) Heatmap of the correlations among the top 50 differential metabolites, visualized by “corrplot” R package. The top 50 differential metabolites were screened according to |log2 FC| values from high to low (|log2 FC|> 0.5). FC, fold change (TIF 5043 KB)

Supplementary file4 Figure S4. Network analysis of the top 5 enriched pathways related to the differential metabolites using Metscape. (A) Tryptophan metabolism network. (B) Nicotinate and nicotinamide metabolism network. (C) Purine metabolism network. (D) Steroid hormone biosynthesis network. (E) Histidine metabolism network (TIF 9386 KB)

Supplementary file5 Table S1: Clinical and demographic data of subjects in this study. (DOCX 11 KB)

Supplementary file6 Table S2: Clean data of 16S rDNA sequencing after optimization. (XLS 30 KB)

Supplementary file7 Table S3: Alpha diversity indices of each feces sample. (XLS 27 KB)

Supplementary file8 Table S4: Microbial community structure of each fecal sample. (XLSX 34 KB)

Supplementary file9 Table S5: Results of UPLC-MS analysis through data pre-processing. (XLSX 752 KB)

Supplementary file10 Table S6: Information about the differential metabolites. (XLSX 86 KB)

## Data Availability

The data used to support the findings of this study are available from the corresponding author upon request.
