# Supplementary figures and images for: Identification of Diagnostic Biomarkers for Compensatory Liver Cirrhosis Based on Gut Microbiota and Urine Metabolomics Analyses
Source: Mol Biotechnol. 2023 Oct 24;66(11):3164–81. doi: 10.1007/s12033-023-00922-9 (PMC11549169; doi:10.1007/s12033-023-00922-9)

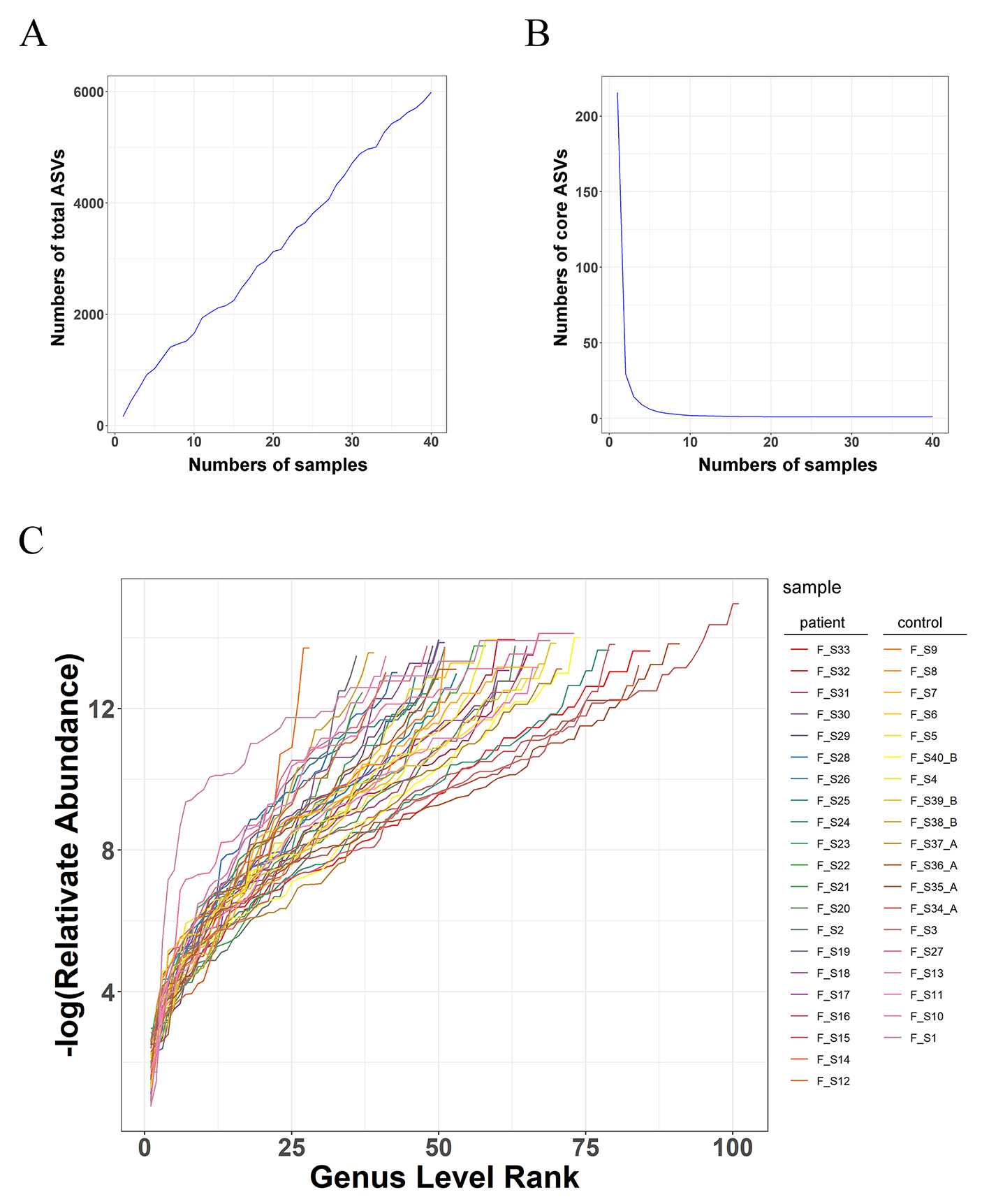

Supplement: Supplementary file 1 — Supplementary file1 Figure S1. Analyses of 16S rDNA sequencing data. (A) Pan-genome analysis of 16S rDNA sequencing on ASV level. (B) Core-genome analysis of 16S rDNA sequencing on ASV level. (C) Rank-abundance curve on genus level. Analyses were performed using “ggplot2” R package. Control group, n = 19; patient group, n = 21. rDNA, ribosomal DNA; ASV, amplicon sequence variant (TIF 8037 KB) [file 12033_2023_922_MOESM1_ESM.tif]

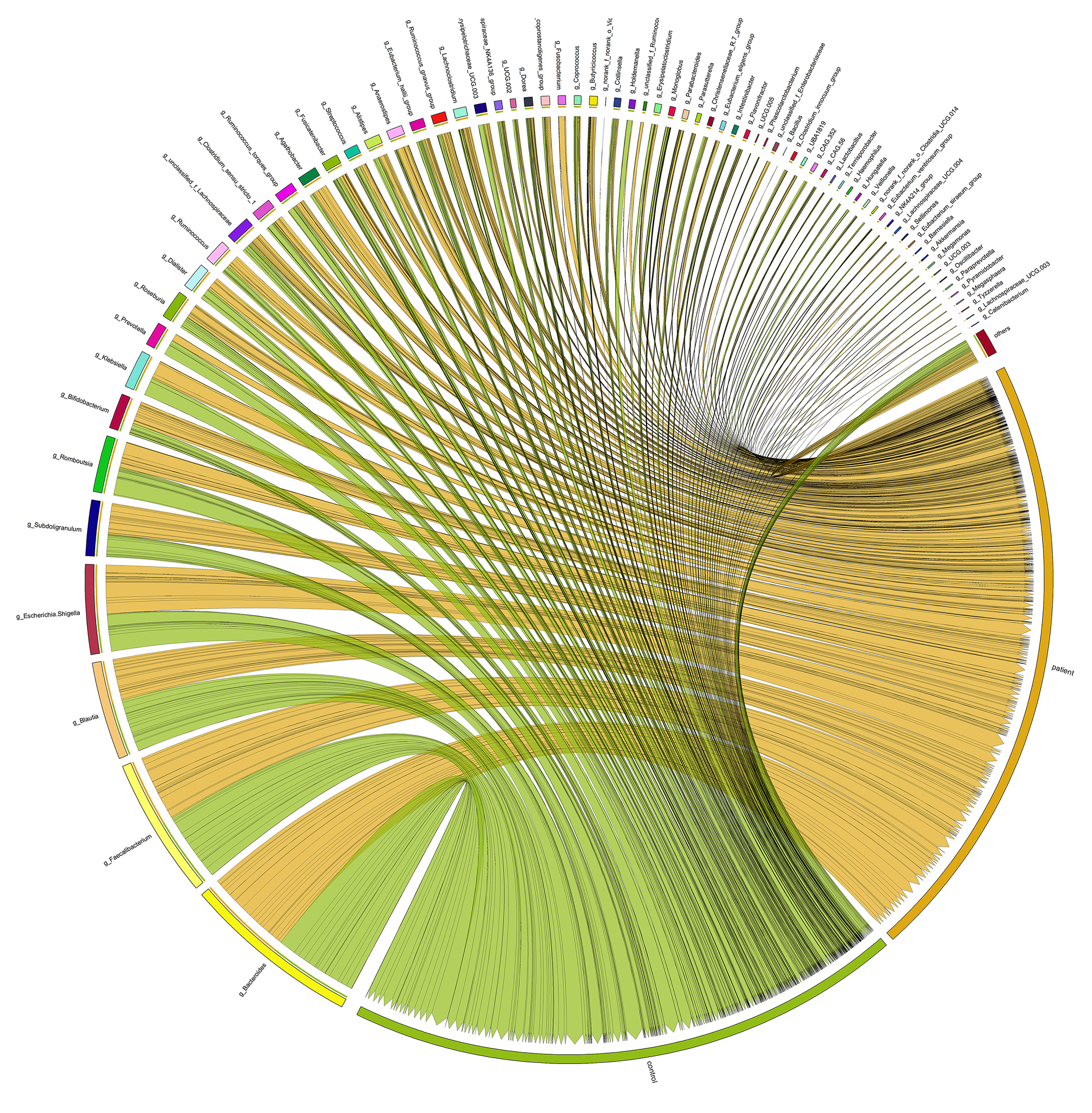

Supplement: Supplementary file 2 — Supplementary file2 Figure S2. Circos diagram of the microbial community composition of each control and patient sample using “RCircos” R package. Control group, n = 19; patient group, n = 21 (TIF 6067 KB) [file 12033_2023_922_MOESM2_ESM.tif]

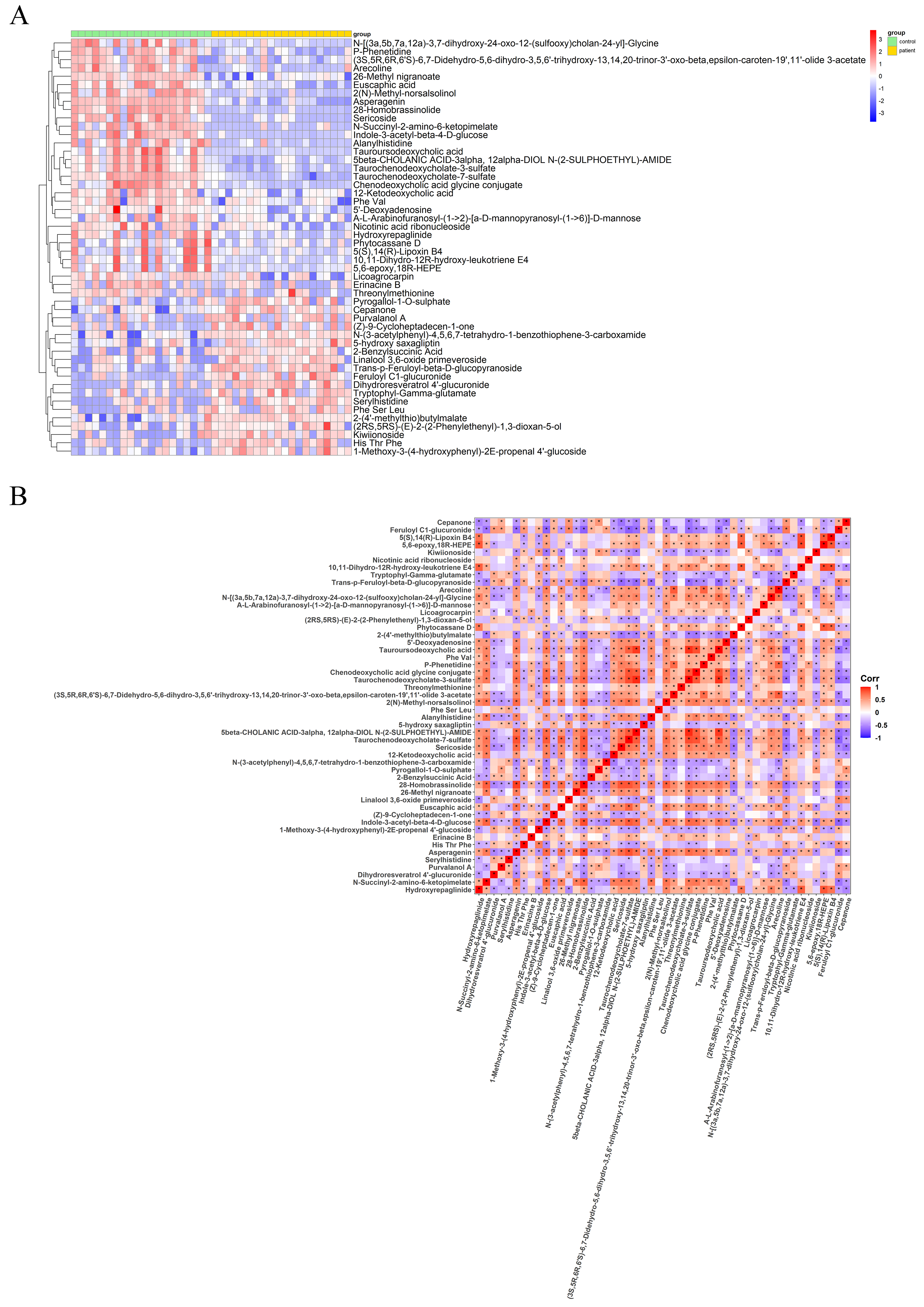

Supplement: Supplementary file 3 — Supplementary file3 Figure S3. Analyses of the top 50 differential metabolites. (A) Heatmap of the distribution of the top 50 differential metabolites in each control and patient sample, visualized by “pheatmap” R package. Control group, n = 20; patient group, n = 20. (B) Heatmap of the correlations among the top 50 differential metabolites, visualized by “corrplot” R package. The top 50 differential metabolites were screened according to |log2 FC| values from high to low (|log2 FC|> 0.5). FC, fold change (TIF 5043 KB) [file 12033_2023_922_MOESM3_ESM.tif]

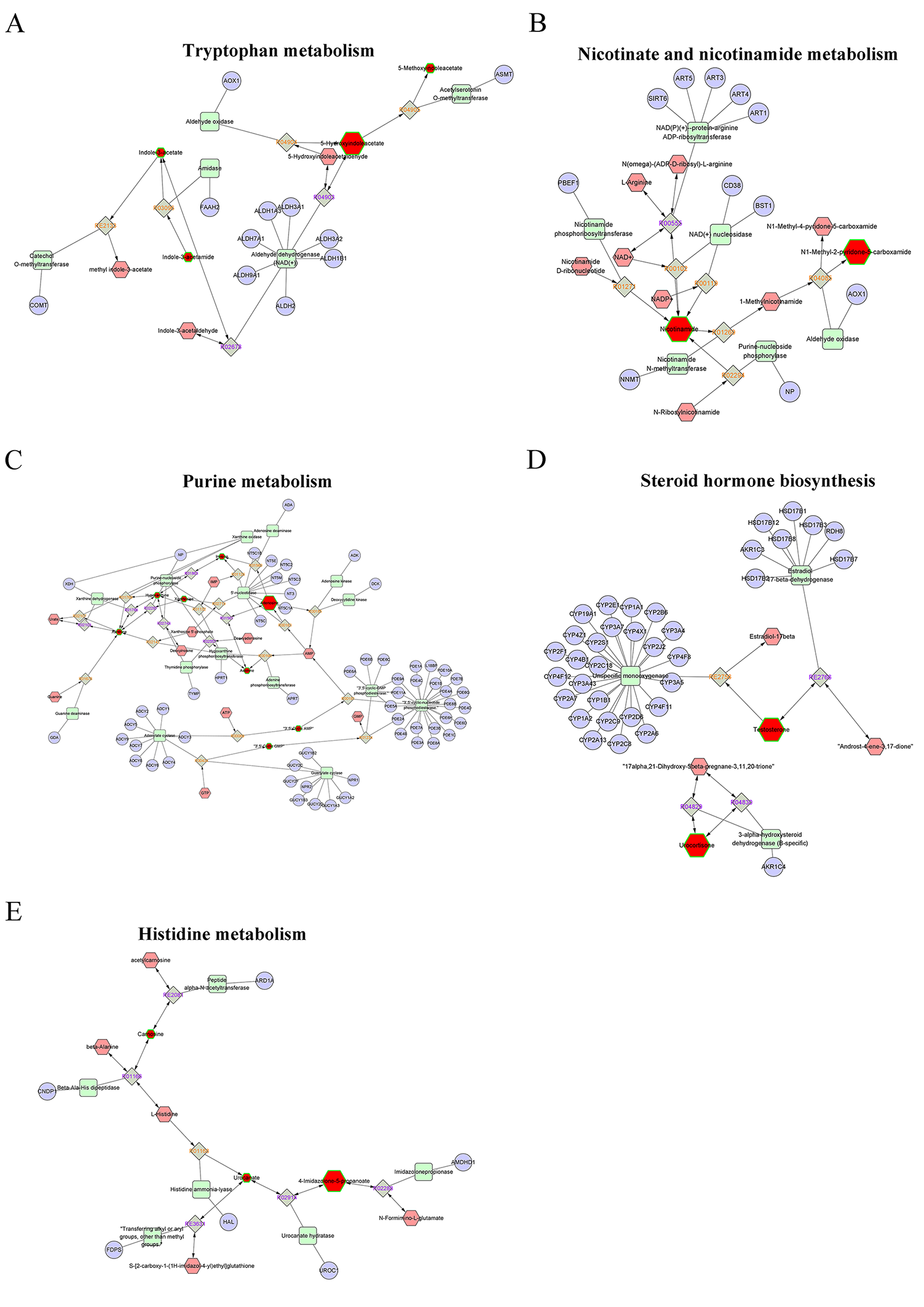

Supplement: Supplementary file 4 — Supplementary file4 Figure S4. Network analysis of the top 5 enriched pathways related to the differential metabolites using Metscape. (A) Tryptophan metabolism network. (B) Nicotinate and nicotinamide metabolism network. (C) Purine metabolism network. (D) Steroid hormone biosynthesis network. (E) Histidine metabolism network (TIF 9386 KB) [file 12033_2023_922_MOESM4_ESM.tif]
