# Supplementary material for: Identification of Diagnostic Biomarkers for Compensatory Liver Cirrhosis Based on Gut Microbiota and Urine Metabolomics Analyses
Source: Mol Biotechnol. 2023 Oct 24;66(11):3164–81. doi: 10.1007/s12033-023-00922-9 (PMC11549169; doi:10.1007/s12033-023-00922-9)
Supplement: Supplementary file 5 — Supplementary file5 Table S1: Clinical and demographic data of subjects in this study. (DOCX 11 KB) [file 12033_2023_922_MOESM5_ESM.docx]

|  | CLC (n = 21) | Healthy ( n = 20) | *P* value |
| --- | --- | --- | --- |
| Gender (male/female) | 14/7 | 10/10 |  |
| Age (year) | 56.52 ± 7.61 | 54 ± 8.21 | 0.444 |
| BMI (kg/m^2^) | 24.14 ± 3.12 | 24.30 ± 2.93 | 0.865 |

Table S1. Clinical and demographic data of subjects in this study.
